# Optimized Stereo‐Electroencephalography‐Guided Three‐Dimensional Radiofrequency Thermocoagulation for Hypothalamic Hamartomas‐Related Epilepsy: A Single‐Center Experience in 69 Patients

**DOI:** 10.1111/cns.70462

**Published:** 2025-06-07

**Authors:** Yang Dai, Yihe Wang, Zesheng Li, Xiaotong Fan, Liankun Ren, Josemir W. Sander, Penghu Wei, Yongzhi Shan, Guoguang Zhao

**Affiliations:** ^1^ Department of Neurosurgery, Xuanwu Hospital Capital Medical University Beijing China; ^2^ Clinical Research Center for Epilepsy Capital Medical University Beijing China; ^3^ Department of Clinical & Experimental Epilepsy UCL Queen Square Institute of Neurology London UK; ^4^ Department of Neurology, Xuanwu Hospital Capital Medical University Beijing China; ^5^ National Clinical Research Center for Geriatric Diseases Beijing China

**Keywords:** epilepsy, neurosurgery, radiofrequency thermocoagulation, stereo‐electroencephalography, treatment

## Abstract

**Background:**

The high risk of resection surgery for hypothalamic hamartoma (HH) epilepsy drives interest in minimally invasive treatment. Stereo‐electroencephalography‐guided three‐dimensional radiofrequency thermocoagulation (SEEG‐3D RFTC) offers an alternative option. We investigated this technology's efficacy, safety, and prognostic risk factors.

**Methods:**

Patients with HH who underwent SEEG‐3D RFTC were retrospectively analyzed. A high‐density focal stereo‐array electrode implantation was adopted. SEEG‐3D RFTC was performed between two contiguous contacts of the same electrode or adjacent contacts of different electrodes. Outcomes were separately evaluated for clinical seizures, gelastic seizures (GS), and non‐gelastic seizures (nGS). Kaplan–Meier survival analysis was used to assess treatment effectiveness. Risk factors were analyzed using log‐rank tests and Cox regression analyses.

**Results:**

Sixty‐nine patients were enrolled. The mean follow‐up was 41.00 ± 18.19 months. Seizure freedom was obtained by 48/69 (69.57%) patients for clinical seizures, 50/62 (80.65%) patients for GS, and 41/54 (75.93%) patients for nGS. Surgical procedures were well tolerated. In this study, the proportion of patients experiencing long‐term complications was 10.14%. The percentages of HH ablation (*p* = 0.003; hazard ratio 0.956, 95% confidence interval 0.928–0.985) and HH attachment ablation (*p* = 0.001; hazard ratio 0.931, 95% confidence interval 0.892–0.970) were significantly associated with seizure outcomes.

**Conclusions:**

Optimized SEEG‐3D RFTC is an effective and safe option for HH‐related epilepsy and is especially suitable for use where laser interstitial thermal therapy is unavailable. Complete ablation of the HH and attachment site is essential for good outcomes.

## Graphical abstract

This study presents the largest cohort to date of SEEG‐3D RFTC treatment for HH‐related epilepsy, demonstrating it as a safe and effective option. Among 69 patients, 69.57% achieved overall seizure freedom, with GS‐free and nGS‐free rates of 80.65% and 75.93%, respectively (mean follow‐up: 41.0 ± 18.2 months). Complete ablation of the HH and its attachment site was key to better seizure outcomes.

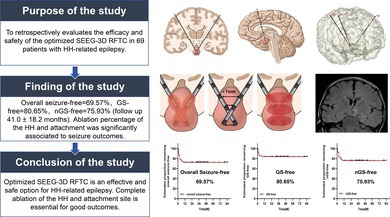

## 1 Introduction

Hypothalamic hamartoma (HH) is a rare congenital non‐neoplastic lesion with an estimated incidence of 1/50,000–1/100,000. It typically presents in childhood, with epilepsy, precocious puberty, and cognitive dysfunction as its most common and characteristic symptoms. Seizures associated with HH are frequently characterized by gelastic seizures (GS) and are often accompanied by other seizure types (nGS), including focal and/or generalized seizures [1]. Since the seizures caused by HH are quite drug‐resistant, surgical treatment is considered imperative for controlling seizures associated with HH. However, achieving an effective and safe disconnection of deep‐seated HHs from the normal hypothalamus remains a significant challenge [2, 3].

Open surgery has traditionally been the primary approach for HH treatment; however, despite various surgical approaches, the complication rate remains high, exceeding 30% [4, 5, 6]. In contrast, minimally invasive techniques such as laser interstitial thermal therapy (LITT), thermocoagulation, and Gamma Knife radiosurgery offer less invasiveness and have increasingly become the first‐line treatment for HH.

Previous studies have examined the efficacy of these techniques. Gamma Knife Surgery (GKS) has demonstrated long‐term outcomes in epilepsy control with a favorable safety profile; however, only 39.6% of patients achieved Engel I outcomes [7]. LITT has shown favorable outcomes in seizure control, whereas large‐scale long‐term cohort studies have not yet been published [8, 9]. Stereo‐electroencephalography‐guided radiofrequency thermocoagulation (SEEG‐RFTC) is also a minimally invasive approach and has been widely applied in epilepsy surgery. Our team reported the first primary evidence supporting the efficacy of SEEG‐RFTC in HH [10]. However, it is widely acknowledged that the limited ablation volume of conventional RFTC protocols constrains its efficacy in seizure control [11]. we optimized the SEEG implantation and RFTC procedure by employing cross‐SEEG electrodes with a three‐dimensional (3D) RFTC protocol [12]. Our previous in vitro experiments suggested that 3D RFTC can achieve 1.81–2.12 times the ablation volume compared to the conventional protocol. This optimized SEEG‐3D RFTC has been successfully employed in various types of epilepsy, resulting in favorable seizure outcomes [13, 14]. In this study, we retrospectively analyzed patients with HH who were treated using the same optimized protocol.

To our knowledge, this represents the largest cohort of patients with HH‐related epilepsy treated using optimized SEEG‐3D RFTC. We analyzed the long‐term seizure outcomes, performed a separate evaluation of seizure remission in GS and nGS, and identified risk factors associated with surgical prognosis. This study aims to provide an effective and technically feasible minimally invasive treatment option for patients with HH‐related epilepsy.

## 2 Methods

### 2.1 Patients

This retrospective study included all patients with HH epilepsy who were treated by SEEG‐3D RFTC between July 2015 and June 2021 at Xuanwu Hospital of Capital Medical University, Beijing, China. Written informed consent was obtained from the patient for publication of this case report and accompanying images. Clinical factors included sex, age, epilepsy duration, precocious puberty (PP), HH classification, HH volume, HH size (maximum diameter in any dimension), and previous surgery. Surgical factors included ablation volume, percentage of HH ablation, and percentage of HH attachment ablation. Cases will be excluded if they meet any of the following criteria: (1) the epilepsy medical records lack confirmed information on disease progression; (2) there is any history of prior neurosurgical or radiosurgical treatments; (3) high‐resolution magnetic resonance imaging (MRI) data required for anatomical analysis is unavailable; (4) MRI analysis reveals the presence of other brain abnormalities in the patient. This study was approved by the Xuanwu Hospital Ethics Committee and conducted in accordance with ethical standards, including the Helsinki Declaration.

### 2.2 Presurgical Workup

**FIGURE 1:**
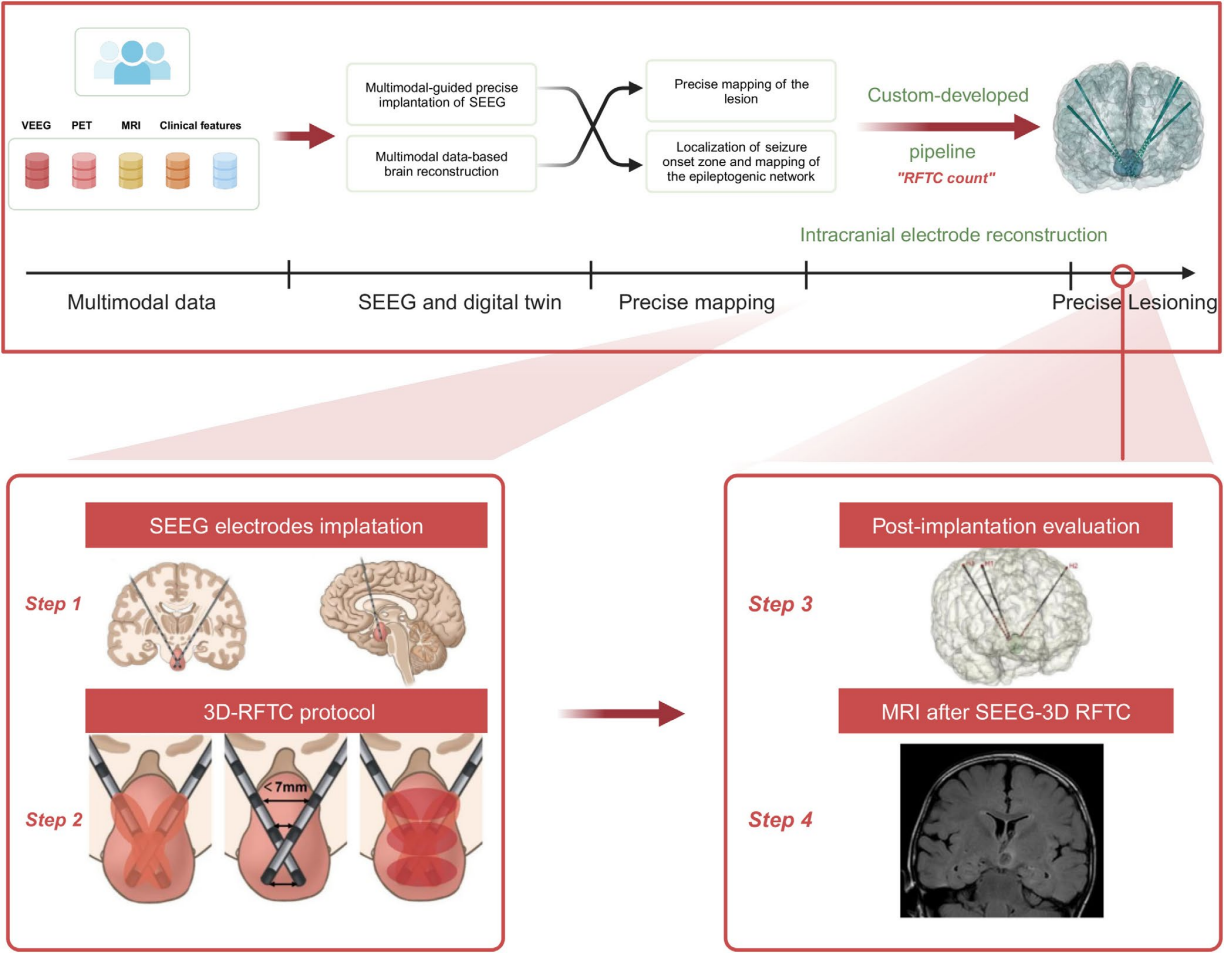
The workflow of optimized SEEG‐3D RFTC. TOP: General workflow of optimized SEEG‐3D RFTC. MDT, multidisciplinary team; SOZ, seizure onset zone. Bottom: Optimized SEEG‐3D RFTC for HH epilepsy. Schematic illustration of the protocol of stereo‐crossed SEEG electrodes implantation in HH. SEEG electrodes were implanted into the HH through a paraventricular approach. Step 1: Coronal and sagittal views of the electrode implantation protocol. Step 2: 3D RFTC protocol. Bipolar coagulation on each of two contiguous contacts in each electrode and 3D bipolar coagulation of contacts in crossover SEEG electrodes with a distance of < 7 mm. Step 3: Post‐implantation evaluation of SEEG electrodes targeting the HH via MRI reconstruction. Step 4: MRI after SEEG‐3D RFTC. SEEG‐3D RFTC, stereo‐electroencephalography‐guided three‐dimensional radiofrequency thermocoagulation; HH, hypothalamic hamartoma; MRI, magnetic resonance imaging. Created in BioRender.com.

All underwent a thorough assessment consisting of video‐electroencephalogram (EEG) monitoring and various neuroimaging exams, including 3.0‐T head MRI and positron emission tomography scans. Based on the coronal MRI scans, HH was categorized as intrahypothalamic, parahypothalamic, mixed with unilateral attachment, or mixed with bilateral attachment [15]. The results of the examination were presented to a multidisciplinary team for further discussion. Strategies for SEEG electrode implementation were devised after the diagnosis of HH‐related epilepsy and the decision to undergo SEEG‐3D RFTC surgery. The workflow of this study is shown in Figure 1.

### 2.3 Implantation of SEEG Electrodes and Intracranial EEG Monitoring

Individualized electrode implantation was designed for people who underwent SEEG‐3D RFTC. Enhanced thin‐layer MRI (slice thickness, 1 mm) and thin‐layer CT (slice thickness, 1 mm) were performed prior to trajectory planning. Images were imported into the Robotized Stereotactic Assistant system (ROSA, Medtech, Montpellier, France) to generate a multimodal fusion that visualized vascular and critical structures, helping avoid vascular injury.

Under general anesthesia, the patient's head was fixed in a stereotactic frame. The robotic arm of the ROSA system guided the electrode holder to each preplanned trajectory, and the surgeon sequentially inserted the electrodes through burr holes. The electrodes (Alcis, Besancon, France) were placed using the ROSA system. Each SEEG electrode consisted of 5–15 cylindrical contacts made of platinum–iridium alloy, with each contact measuring 2 mm in length and 0.8 mm in diameter. The center‐to‐center spacing between contacts was 3.5 mm.

Trajectories were individually designed to optimize sampling from the HH and its anatomical attachments. The paraventricular approach was preferred to achieve broader access to the lesion and its interface with the third ventricle. The lesion border was defined based on signal intensity differences between the HH and the ventricular wall on thin‐layer MRI. Each trajectory passed through the superior or middle frontal gyrus and terminated at the medial edge of the HH.

Multiple electrodes were implanted bilaterally, with a focus on covering the internal structure of the hamartoma. The implantation strategy was to ensure that the distance between electrode targets was less than 7 mm, so that overlapping ablation fields could be formed between adjacent electrode contacts. This design allowed for high‐density sampling and effective thermal lesioning. A postoperative CT was performed within 24 h to verify electrode positioning. SEEG monitoring was initiated after confirming accuracy and excluding complications such as hemorrhage.

### 2.4 Application Accuracy

The exact contact locations of implanted electrodes were verified using postoperative 3D CT co‐registered with preoperative 3D T1‐weighted MRI. The preoperative planning trajectory imaging data in the robot system were integrated with the postsurgical CT. The entry point was defined as the center position where the electrode trajectory entered the cortex, and the target was defined as the final position of the electrode tip. The planned and the actual trajectory were determined from the coronal, sagittal, and transverse planes. The unsigned Euclidean distance between the planned and the actual entry point (entry point localization error [EPLE]) and between the planned and the actual target point (target point localization error [TPLE]) were measured using the distance measurement function in the robot software system.

### 2.5 RFTC Count Pipeline

To enhance the precision of electrode reconstruction, we have developed the RFTC Count pipeline. RFTC Count is a pipeline designed to process and analyze electrode contact data to filter out candidate contact pairs that meet specific distance criteria, aiming to provide a reference for RFTC treatment. The specific steps are as follows: (1) Calculate entry and target point coordinates. Based on the voxel size input by the user and the number of electrode contacts on each electrode, calculate the three‐dimensional coordinates of the entry point and the target point for each electrode. (2) Determine precise coordinates of each contact. For each electrode, use the coordinates of the entry and target points to evenly divide the electrode according to the number of contacts. This allows for the accurate calculation of the precise coordinates of each contact.

### 2.6 3D RFTC Surgery and Ablation Lesion Evaluation

The selection of ablation electrode contacts was based on preoperative assessment and intracranial EEG monitoring. The inclusion criteria were (1) sites of epileptogenic initiation during seizure onset or significant epileptic discharge during interictal periods within the HH, and (2) preoperative assessment of contacts designed to be located at the HH attachment site. Contacts located < 2 mm from the vessel were excluded.

The surgery was performed in an awake state, and the corresponding contacts were connected to an R200BM1 RFTC generation system (Beijing Neo Science Co., Beijing, China). Ablation was performed as bipolar ablation first with a pre‐ablation pulse of 2 W for 40 s, followed by two pulses of 3 W for 60 s between two adjacent contacts on the same electrode. Then, 3D ablation was carried out on contacts with distances < 7 mm on parallel and cross electrodes for two pulses of 3 W for 60 s. Intracranial EEG was recorded for 10 min following ablation. Guiding screws and electrodes were extracted after ensuring no abnormalities or intracranial discharges.

**FIGURE 2:**
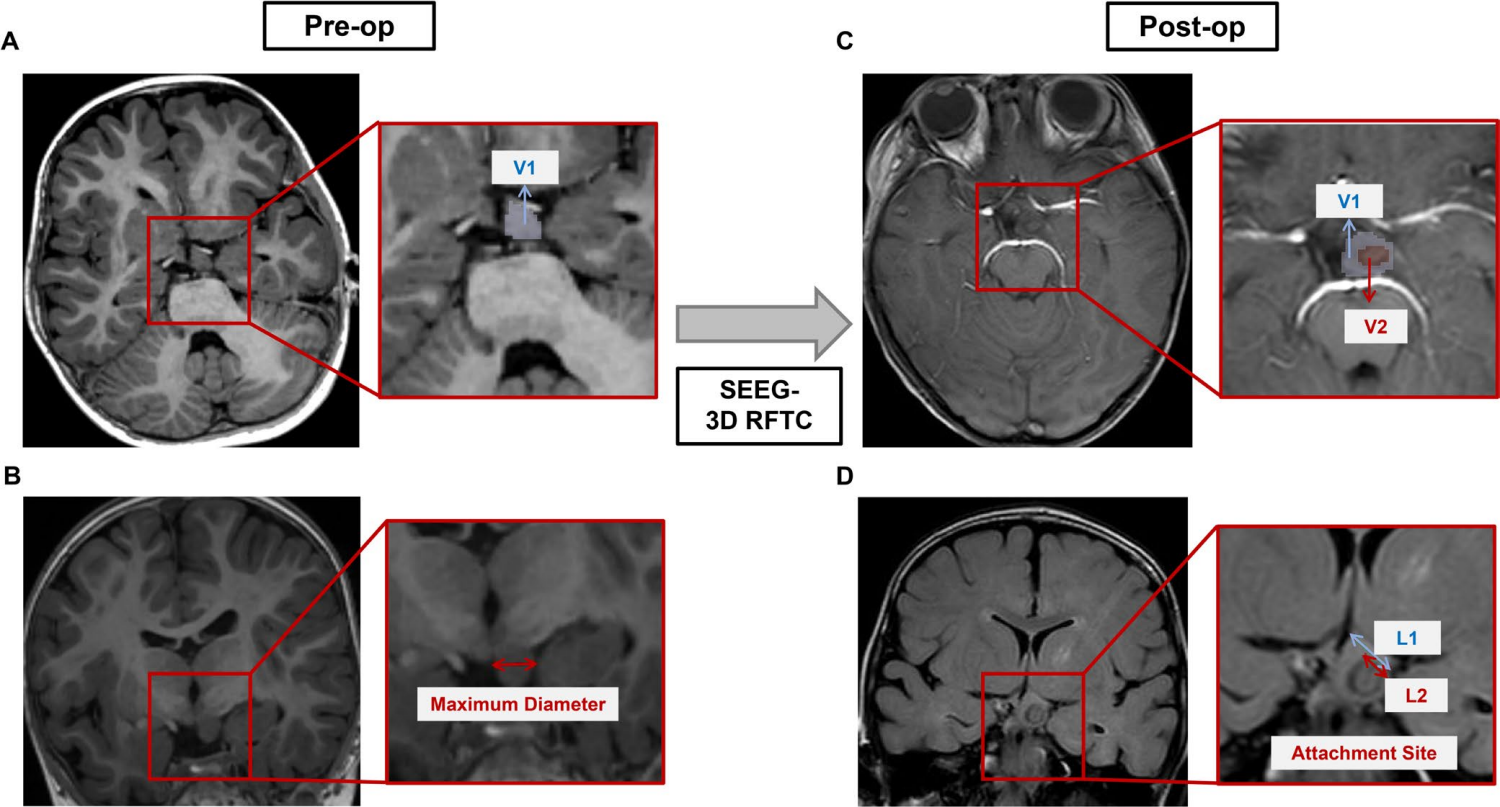
Preoperative measurement of HH and postoperative measurement of 3D RFTC lesion. (A) Preoperative axial view of HH. The partial magnification outlines the range of the HH. (B) Preoperative coronal view of HH. The partial magnification shows the maximum diameter of HH. (C) Postoperative axial view of HH. The partial magnification outlines the range of the ablation and the residual HH. V1 = The measured volume of HH, V2 = The measured volume of 3D RFTC lesion in HH. (D) Postoperative coronal view of HH. The partial magnification shows the attachment of HH and the 3D RFTC range of the attachment. L1 = The maximal attachment between the HH and the hypothalamus, L2 = The 3D RFTC range in HH attachment. HH, hypothalamic hamartoma; SEEG‐3D RFTC, stereo‐electroencephalography‐guided three‐dimensional radiofrequency thermocoagulation.

3D RFTC lesion volume was evaluated using postoperative MRI data processed in 3D Slicer software (version 4.10.2). The ablation lesion was manually segmented slice‐by‐slice to delineate the boundaries; the reconstruction was then generated, and the lesion volume was automatically computed. The percentage of HH ablation was calculated by comparing the 3D RFTC lesion volume with the preoperatively measured HH volume, which was segmented and reconstructed using the same method. The percentage of HH attachment ablation was assessed on the MRI slice demonstrating the maximal interface between the HH and the hypothalamus (Figure 2).

### 2.7 Follow‐Up and Assessment of Seizure Outcomes

Outcomes in terms of seizure suppression and surgery‐related complications were evaluated at 6‐month intervals following the procedure. Seizure outcomes were evaluated separately for clinical seizures, GS, and nGS, according to the League Against Epilepsy (ILAE) outcome classification [16]. ILAE I was considered seizure‐free.

### 2.8 Statistical Analysis

Statistical analyses were performed using SPSS 26 (IBM, Armonk, NY). The duration to the first occurrence of postoperative seizure was set as the endpoint for Kaplan–Meier survival analysis. Additionally, seizure‐free survival was analyzed for each investigated factor. Categorical variables were compared using the log‐rank test. Continuous variables, including age at surgery, age at seizure/GS/nGS onset, and epilepsy duration, were dichotomised along the median [17]. HH size was dichotomised using a maximum diameter of 15 mm [18]. The percentages of HH ablation and HH attachment ablation underwent univariate Cox analysis as continuous variables. Variables with *p* < 0.2 in univariate analyses were included in the multivariate Cox regression analysis. Statistical significance was established at *p* < 0.05.

## 3 Results

### 3.1 Patient Characteristics

Sixty‐nine patients (44 males and 25 females) who underwent SEEG‐3D RFTC were included, among which 62 had GS and 54 had nGS. The ages at surgery and seizure onset, as well as the duration of epilepsy were 10.58 ± 8.85, 3.37 ± 3.83, and 7.24 ± 7.17 years, respectively. For GS patients, the ages at surgery and GS onset, as well as the duration of GS were 9.84 ± 8.12, 2.94 ± 3.18, and 6.79 ± 7.02 years, respectively. For nGS patients, the ages at surgery and nGS onset, as well as the duration of nGS were 11.74 ± 9.36, 7.07 ± 5.80, and 4.10 ± 5.22 years, respectively (Table 1).

**TABLE 1:** SEEG‐3D RFTC patients' profiles and seizure outcomes.

Total	69
Sex (male: female)	44: 25
Age at surgery (years)	10.58 ± 8.85
Age at seizure onset (years)	3.37 ± 3.83
Duration of seizure (years)	7.24 ± 7.17
GS	62 (89.86%)
Age at GS onset (years)	2.94 ± 3.18
Duration of GS (years)	6.79 ± 7.02
nGS	54 (78.26%)
Age at nGS onset (years)	7.07 ± 5.80
Duration of nGS (years)	4.10 ± 5.22
Volume of HHs (cm^3^)	2.03 ± 1.69
Maximum diameter	
≤ 15 mm	42 (60.87%)
> 15 mm	27 (39.13%)
HH subtypes	
Parahypothalamic	15 (21.74%)
Intrahypothalamic	16 (23.19%)
Mixed, unilateral	15 (21.74%)
Mixed, bilateral	23 (33.33%)
Seizure frequency	
Daily	37 (53.62%)
Weekly	12 (17.39%)
Monthly	20 (28.99%)
Precocious puberty	14 (20.29%)
Previous surgery	20 (28.99%)

Abbreviations: GS, gelastic seizures; HH, hypothalamic hamartoma; nGS, non‐gelastic seizures; SEEG‐3D RFTC, stereo‐electroencephalography‐guided three‐dimensional radiofrequency thermocoagulation.

The maximum diameter of HH was < 15 mm in 42 patients and > 15 mm in 27 patients. The mean volume of the HH was 2.03 ± 1.69 cm [3]. Mixed type with bilateral attachment was the most observed subtype, accounting for 33.33% (23/69), followed by intrahypothalamic type (23.19%, 16/69), mixed type with unilateral attachment (21.74%, 15/69), and parahypothalamic type (21.74%, 15/69).

### 3.2 SEEG Implantation and 3D RFTC Characteristics

**FIGURE 3:**
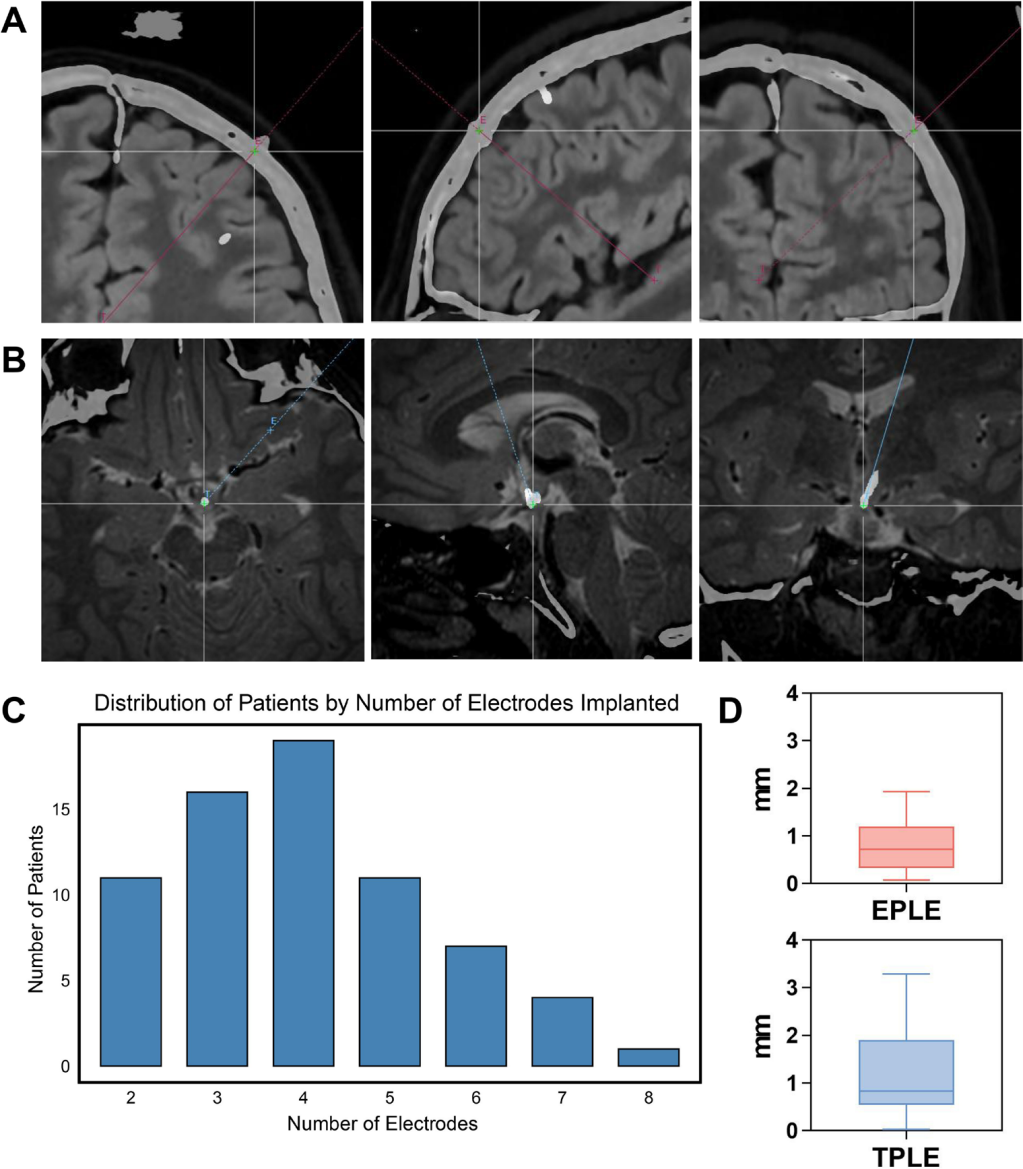
SEEG electrode implantation characteristics. (A) Determination of EPLE. Axial, sagittal, and coronal views of planned and actual electrode trajectories at the entry point. (B) Determination of TPLE. Axial, sagittal, and coronal views of planned and actual electrode trajectories at the target point. (C) Distribution of patients by number of SEEG electrodes implanted. (D) The EPLE and TPLE of SEEG electrodes in this study. EPLE = 0.81 ± 0.51 mm and TPLE = 1.15 ± 0.82 mm. SEEG, stereo‐electroencephalography; EPLE, entry point localization error; TPLE, target point localization error.

In this study, all electrode implantations targeted the HH region. A total of 279 electrodes were implanted, with 2–8 electrodes per implantation. The entry point and target point errors of all electrodes were statistically analyzed. TPLE was 1.15 ± 0.82 mm and EPLE was 0.81 ± 0.51 mm. The distribution of the number of implanted electrodes and accuracy error of SEEG are shown in Figure 3.

**FIGURE 4:**
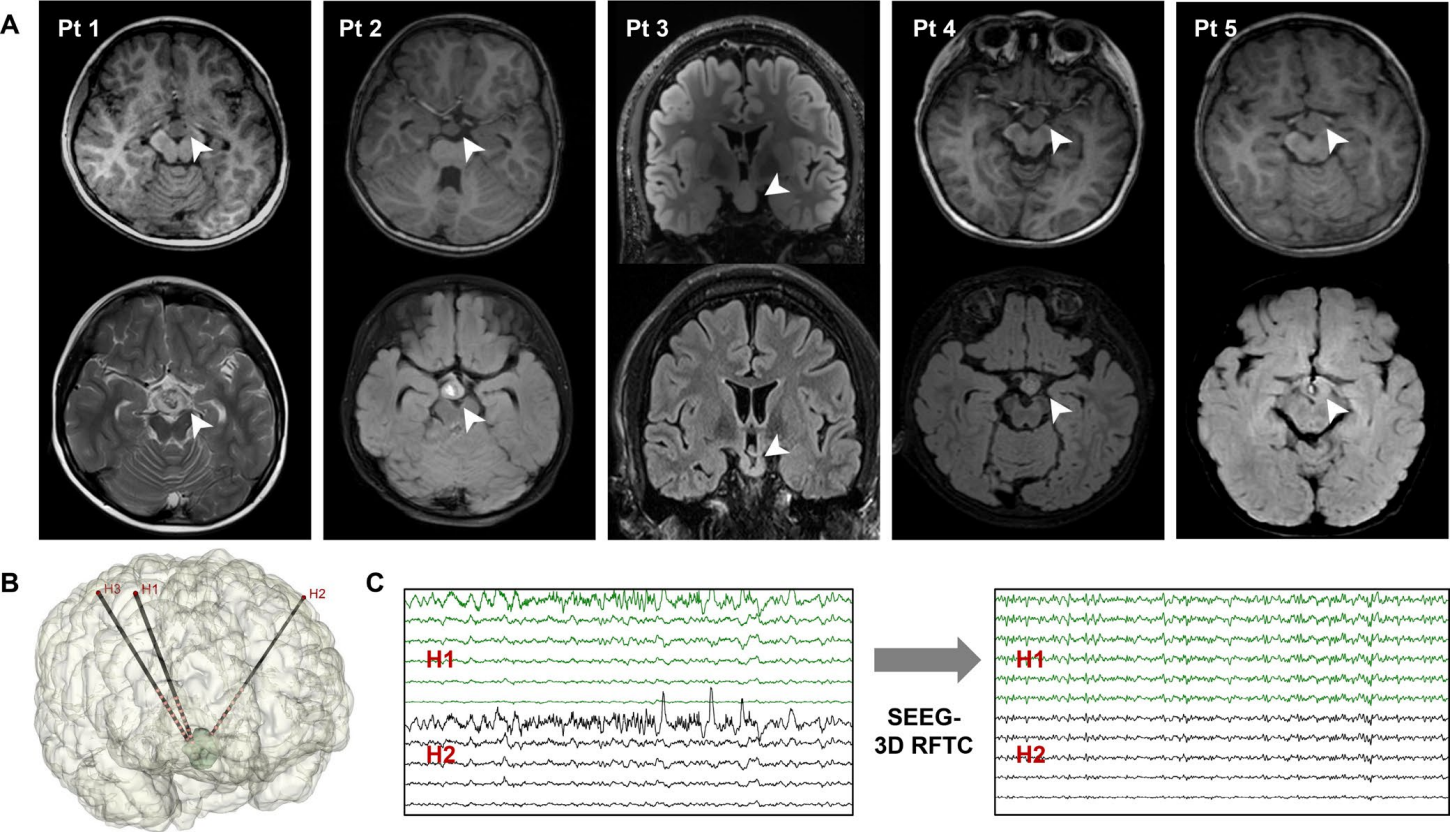
Typical cases of SEEG‐3D RFTC in HH. (A) Pre‐ and post‐operative MRI from five representative patients illustrating the lesion areas created by SEEG‐3D RFTC. (B) Reconstruction of SEEG electrodes in Pt 1. (C) SEEG monitoring before and after 3D RFTC revealed the complete disappearance of epileptiform activity within the HH. SEEG‐3D RFTC, stereo‐electroencephalography‐guided three‐dimensional radiofrequency thermocoagulation; HH, hypothalamic hamartoma; Pt, patient.

The 3D RFTC lesions were reconstructed and volumes were quantified. The mean 3D RFTC volume was 1.39 ± 1.10 cm [3]. Based on the preoperatively measured HH volume, the mean percentage of HH ablation was 72.98% ± 16.49%. The extent of ablation at the HH attachment site reflects the degree of disconnection of the HH; the mean percentage of HH attachment ablation was 63.41% ± 12.01% (Table 2). Typical cases of 3D RFTC are shown in Figure 4.

**TABLE 2:** SEEG‐3D RFTC patients' outcomes.

Follow‐up period (months)	41.00 ± 18.19
Volume of 3D RFTC ablation (cm^3^)	1.39 ± 1.10
Ablation ratio (%)	
Percentage of HH ablation	72.98 ± 16.49
Percentage of HH attachment ablation	63.41 ± 12.01
SEEG‐3D RFTC‐related major complications	
Weight gain	3 (4.35%)
Hypothyroidism	1 (1.45%)
Final seizure outcomes	
Overall seizure‐free	69.57% (48/69)
GS‐free	80.65% (50/62)
nGS‐free	75.93% (41/54)

Abbreviations: GS, gelastic seizures; HH, hypothalamic hamartoma; nGS, non‐gelastic seizures; SEEG‐3D RFTC, stereo‐electroencephalography‐guided three‐dimensional radiofrequency thermocoagulation.

### 3.3 Seizure Outcomes

**FIGURE 5:**
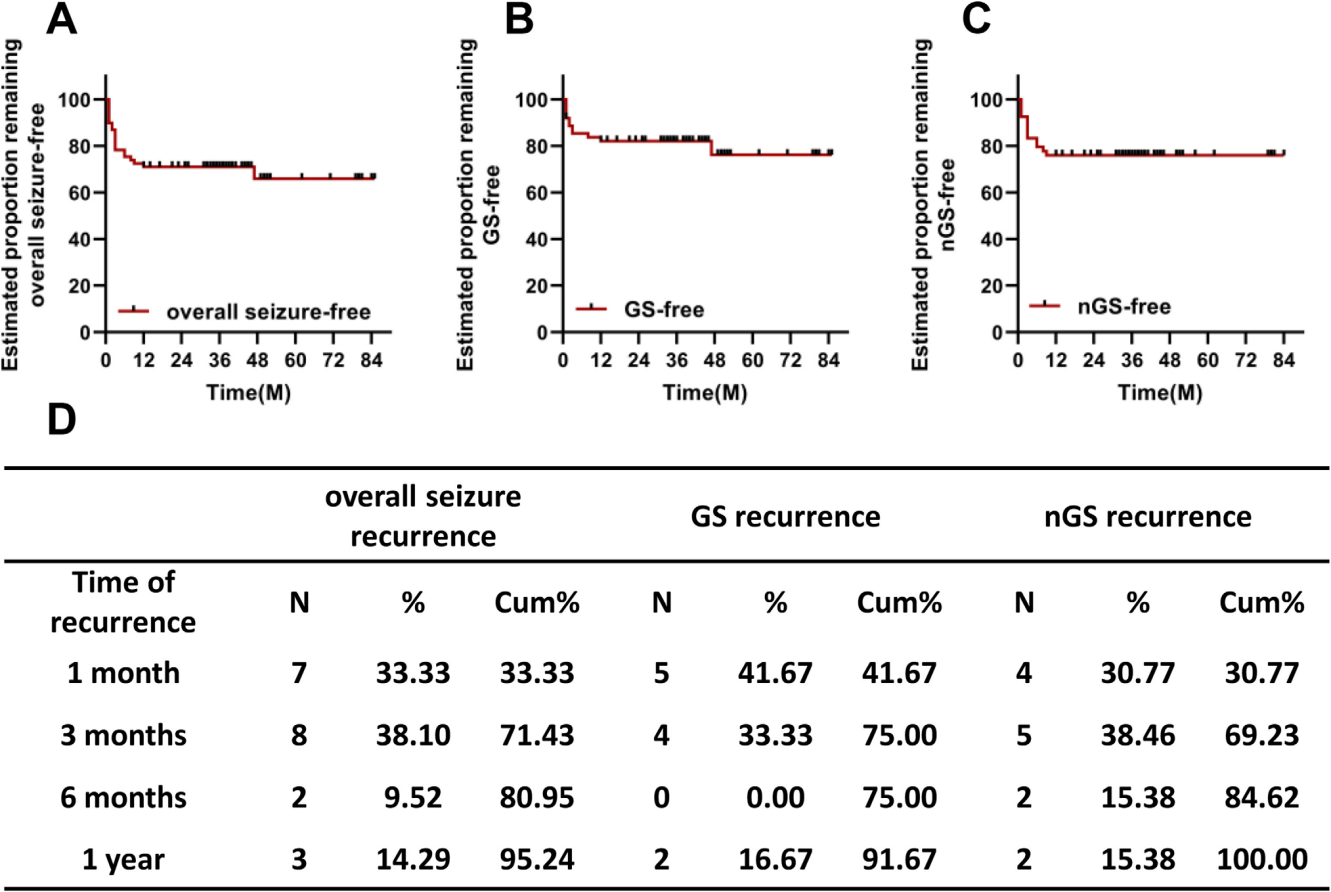
Kaplan–Meier survival curve analysis after SEEG‐3D RFTC. (A) Overall seizure‐free, (B) GS‐free, and (C) nGS‐free survival curves after SEEG‐3D RFTC. (D) The table lists recurrence numbers and rates at each timepoint. GS, gelastic seizures; nGS, non‐gelastic seizures; SEEG‐3D RFTC, stereo‐electroencephalography‐guided three‐dimensional radiofrequency thermocoagulation; Cum, cumulative.

The mean follow‐up was 41.00 ± 18.19 months (12–85 months). For all patients, 69.57% (48/69) were seizure‐free at the end of follow‐up, with a mean seizure‐free survival time of 59.45 ± 4.69 months (95% confidence interval [CI], 50.26–68.64 months). Most instances of seizure recurrence (80.95%) occurred within 6 months of surgery. The seizure‐free rates were 89.86%, 78.26%, and 75.36% at 1, 3, and 6 months, respectively, and remained stable throughout follow‐up (Figure 5).

For the 62 GS patients, the mean GS‐free survival time was 68.12 ± 4.43 months (95% CI, 59.43–76.80 months). Of these patients, 91.94%, 85.48%, and 85.48% were GS‐free at 1, 3, and 6 months, respectively. The GS outcome remained favorable, and 80.65% (50/62) were GS‐free by the end of follow‐up (Figure 5).

**FIGURE 6:**
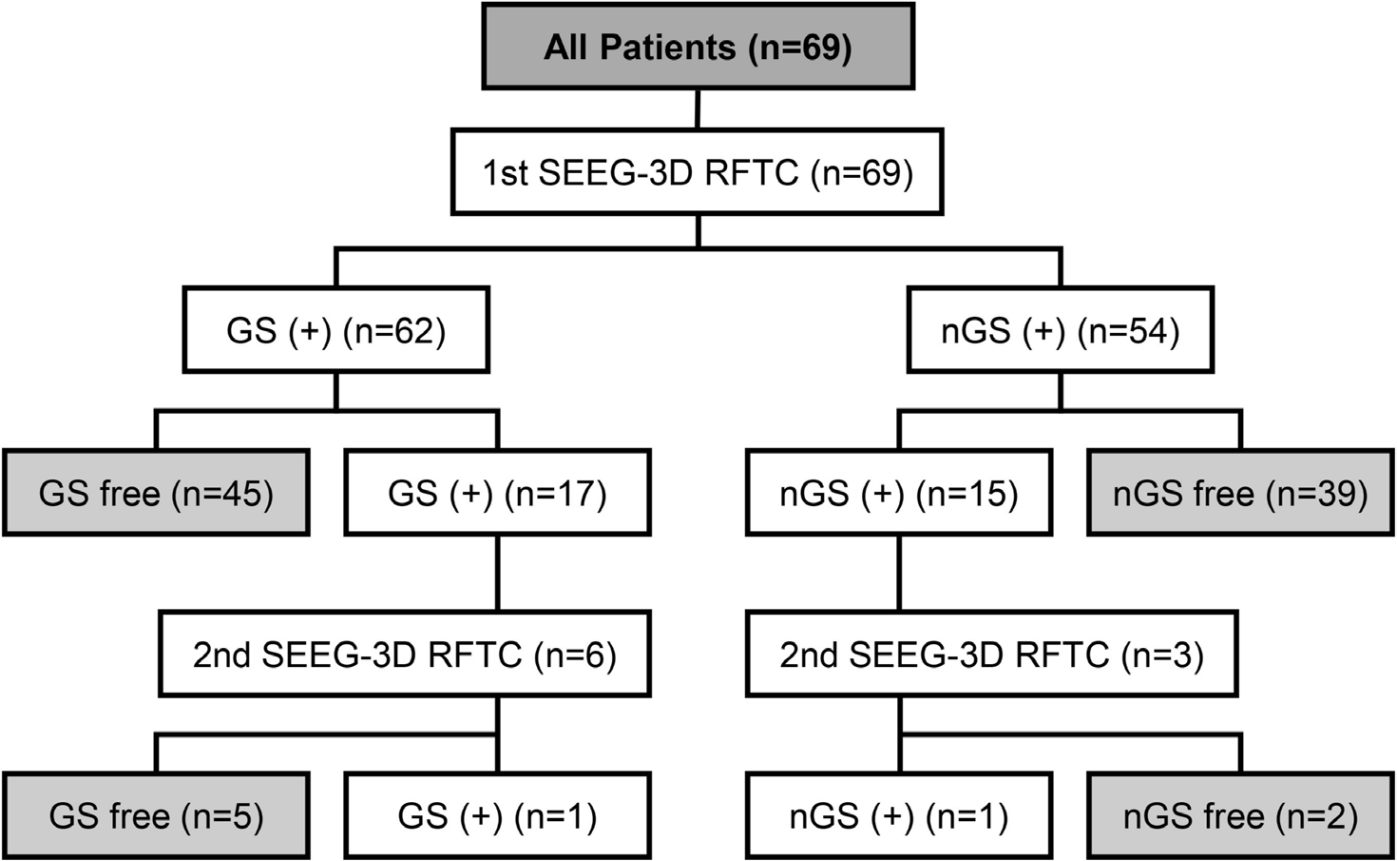
GS and nGS outcomes after SEEG‐3D RFTC. Flow diagram showing the repeat surgeries and outcomes of SEEG‐3D RFTC with respect to GS and nGS in 69 patients. Overall GS freedom and nGS freedom were achieved in 50 (80.65%) and 41 (75.93%) patients, respectively. GS, gelastic seizures; nGS, non‐gelastic seizures; SEEG‐3D RFTC, stereo‐electroencephalography‐guided three‐dimensional radiofrequency thermocoagulation.

For the 54 nGS patients, the mean nGS‐free survival time was 64.67 ± 4.68 months (95% CI, 55.50–73.83 months). Of these patients, 92.59%, 83.33%, and 79.63% were nGS‐free at 1, 3, and 6 months, respectively. The nGS outcome also remained stable in the long term, with 75.93% (41/54) being nGS‐free by the end of follow‐up (Figure 5). The procedures and outcomes of patients are shown below (Figure 6).

### 3.4 Surgical Safety

The complications are summarized in Table 2. All patients tolerated the procedure well, with no complications observed in association with SEEG implantation.

After SEEG‐3D RFTC, all patients underwent CT scans within 24 h, revealing low‐density changes in the ablation regions. Despite varying degrees of oedema at the HH attachment site, no significant symptoms were observed, and the oedema gradually resolved.

Complications associated with 3D RFTC were mainly short‐term, seven patients (10.14%) experiencing transient hyperthermia (> 38°C). Two patients (2.90%) had short‐term memory impairment. Four patients (5.80%) experienced diabetes insipidus. These patients achieved complete remission after conservative treatment. Hence, they were considered minor complications.

Complications that necessitated long‐term management were classified as major. Four patients (5.80%) experienced major complications (weight gain [*n* = 3] and hypothyroidism [*n* = 1]) (Table 2).

### 3.5 Risk Factors Related to Seizure Outcomes

For the overall patients with HH epilepsy, univariate analysis demonstrated that previous surgery, the percentage of HH ablation, and the percentage of HH attachment ablation were significantly related to freedom from seizures after SEEG‐3D RFTC (Table S1). The multivariate Cox regression model revealed that the percentages of HH ablation and HH attachment ablation were significant risk factors (Table 3).

**TABLE 3:** Risk factors of HH epilepsy treated with SEEG‐3D RFTC.

Risk factors	Log‐rank/Univariate Cox	Multivariate Cox		
	*p*	HR	*p*	95% CI
Overall seizure outcomes				
Previous surgery	0.014	1.055	0.924	0.348–3.201
Percentage of HH ablation	< 0.001	0.956	0.003	0.928–0.985
Percentage of HH attachment ablation	< 0.001	0.931	0.001	0.892–0.970
GS outcomes				
Age at GS onset	0.096	1.617	0.590	0.282–9.283
HH size (maximum diameter)	0.079	0.851	0.794	0.254–2.856
Percentage of HH ablation	< 0.001	0.883	< 0.001	0.833–0.937
Percentage of HH attachment ablation	0.001	0.985	0.593	0.930–1.042
nGS seizure outcomes				
Age at surgery	0.003	0.880	0.922	0.067–11.480
Age at nGS onset	0.003	0.280	0.253	0.031–2.487
Duration of nGS	0.049	1.183	0.837	0.238–5.894
Previous surgery	0.002	0.783	0.770	0.153–4.020
HH classification	0.165	0.938	0.831	0.523–1.683
Percentage of HH ablation	0.014	0.992	0.666	0.955–1.030
Percentage of HH attachment ablation	< 0.001	0.887	< 0.001	0.831–0.948

Abbreviations: CI, confidence interval; GS, gelastic seizures; HH, hypothalamic hamartoma; HR, hazard ratio; nGS, non‐gelastic seizures; RCS, restricted cubic spline; SEEG‐3D RFTC, stereo‐electroencephalography‐guided three‐dimensional radiofrequency thermocoagulation.

For GS outcome, the percentage of HH ablation and the percentage of HH attachment ablation were significantly related to freedom from GS according to the univariate analyses (Table S2). However, the multivariate Cox regression model showed that the only significant risk factor for freedom from GS was the percentage of HH ablation (Table 3).

For nGS outcome, age at surgery and nGS onset, duration of nGS, previous surgery, and percentages of HH ablation and HH attachment ablation were significantly associated with freedom from nGS according to the univariate analyses (Table S3). Multivariate Cox regression analysis showed that the percentage of HH attachment ablation was the only significant factor associated with freedom from nGS (Table 3).

## 4 Discussion

In the present study, we used an optimized SEEG‐3D RFTC technology in treating HH and reported the long‐term follow‐up results. Seizure outcomes were evaluated separately for overall seizures, GS, and nGS. Most seizure recurrence after the SEEG‐3D RFTC appeared within 1 year. Prognostic analysis revealed that the percentages of HH ablation and HH attachment ablation are risk factors significantly influencing the overall seizure control.

### 4.1 Effectiveness of SEEG‐3D RFTC in HH Epilepsy

Stereo‐crossed SEEG allowed for high‐density coverage of the HH and its attachment site. This arrangement enabled the investigation of the intricate electrical pathway between the HH and the intercortical epileptic network. It also served as the foundation for the 3D RFTC protocol.

The optimized SEEG‐3D RFTC protocol aimed to disconnect the HH lesion by means of in situ ablation within the HH itself and the attachment site [19]. The advantage of this modified 3D RFTC protocol has been verified by our research team through a comprehensive set of ex vivo and in vivo experiments. These investigations provide evidence that 3D RFTC technology can achieve a maximum ablation distance of 7 mm [12]. In clinical application, this optimized technique has been validated in hippocampal sclerosis, insula epilepsy, and other forms of focal epilepsy. It has demonstrated the total eradication of the epileptogenic focus and achieved satisfactory seizure control outcomes [13, 14]. In the case of HH, most epilepsy cases arise from the tumor itself, and the epileptic focus is well‐defined and confined [20]. Meanwhile, through the utilization of the cross‐designed SEEG electrodes and 3D RFTC, it is feasible to achieve total ablation of the vast majority of HH. Therefore, we believe that patients with HH epilepsy are particularly suitable for SEEG‐3D RFTC.

This study further substantiates our perspective. The overall seizure‐free rate reached 69.57% among 69 patients over an average follow‐up of 41.00 months, indicating the satisfactory and stable therapeutic effect of SEEG‐3D RFTC in treating HH. In comparison with a previous study of LITT, 80% of patients with GS and 56% of patients with nGS achieved seizure freedom [21]; the optimized SEEG‐3D RFTC protocol demonstrated comparable effectiveness in terms of seizure control, with GS‐ and nGS‐free rates of 80.65% and 75.93%, respectively. In comparison with prior research on SEEG‐RFTC, our study also exhibits distinct advantages. Liu et al. attempted to improve the prognosis of SEEG‐RFTC by focusing on the ablation of HH attachment and achieved 70.4% Engel I in 27 patients [22]. Wang et al. adopted a similar cross‐ablation protocol and achieved favorable surgical results in 28 patients [18]. Previous studies provided preliminary evidence of the feasibility of SEEG‐RFTC in HH, and our study verified the superior efficacy of optimized SEEG‐3D RFTC in HH with a larger patient cohort and longer follow‐ups.

SEEG‐3D RFTC also outperformed other surgical treatments in seizure control, including craniotomy and GKS. Previous studies of craniotomy demonstrated that complete resection of HH could not be achieved in 38.5%–65.4% of patients and the seizure‐free rate varies from 15% to 54% [23, 24, 25]. Even though GKS is relatively safe and can be operated in multiple stages, seizure control effectiveness requires improvement. A prospective trial evaluating the safety and efficacy of GKS demonstrated that 39.6% of patients achieved Engel class I outcomes, while 58.3% required a second treatment [7]. The routine use of surgical robots significantly enhances the precision of SEEG implantation, consequently contributing to improved efficacy in ablation and seizure control.

MRI‐stereotactic radiofrequency thermocoagulation (SRT) is another similar ablation option for HH. Kameyama et al. showed that the overall seizure‐free, GS‐free, and nGS‐free rates were 71%, 86%, and 78.9%, respectively [15, 26]. However, neither LITT nor SRT can directly confirm the epileptogenic origin in HH. Clinically, we observed that some HH patients have non‐HH seizure onset, raising concerns about potential overtreatment. In contrast, SEEG‐3D RFTC enables intraoperative electrophysiological confirmation and targeted ablation [26, 27]. Its ability to map real‐time epileptogenic activity offers a key advantage over purely anatomy‐based approaches. Shirozu et al. demonstrated that the electrophysiological border between HH and the hypothalamus closely corresponds to the MRI‐defined boundary, and that the most active epileptogenic zones are near this interface [28]. Furthermore, Scholly et al. reported that in some HH patients, seizures may originate from or evolve to independent neocortical areas through a kindling‐like process of secondary epileptogenesis [29]. SEEG is currently the only tool capable of directly identifying such widespread ictogenic networks. Thus, SEEG‐guided ablation may not only improve targeting accuracy but also prevent under‐ or overtreatment in patients with atypical seizure propagation patterns.

### 4.2 Safety of SEEG‐3D RFTC in HH Epilepsy

In this study, SEEG‐3D RFTC was demonstrated to be safe and practical. Complications related to the procedure were mainly short‐term, including transient hyperthermia, short‐term memory impairment, pituitary dysfunction, and weight gain. Long‐term complications were observed in only four patients. Consistent with previous studies on RFTC and SRT, hyperthermia was the most common complication. However, Wang et al. did not observe hyperthermia in their study [18]. Our study intentionally covered ablation of the HH attachment, and 3D RFTC achieved more extensive damage, which may contribute to heat conduction in the hypothalamus and result in postoperative hyperthermia.

Compared with other surgical interventions, SEEG‐RFTC offers unique advantages, particularly in functional precision and safety. In craniotomy for HH, most patients experience at least transient hyperthermia, and surgical complication rates remain above 30%, even with intraoperative navigation [30]. As a result, minimally invasive approaches such as LITT and SEEG‐3D RFTC have gained increasing attention. LITT, despite its MRI‐guided precision, requires high‐energy delivery, which increases the risk of thermal diffusion [31]. Santiago et al. reported that LITT can cause damage beyond the intended boundary, leading to serious complications such as hemiplegia [32]. Xu et al. found short‐term and long‐term dysfunction rates of 39% and 29%, respectively [21]. Additional risks include memory impairment, delayed hypothyroidism, and hypothalamic obesity, particularly in larger lesions. In contrast, SEEG‐3D RFTC enables stepwise, adjustable ablation guided by real‐time electrophysiological feedback. Lesion size and trajectory can be modified intraoperatively, reducing the risk of unintended injury. Conducted in the awake state and tailored to functional mapping, SEEG‐RFTC enhances safety and adaptability in anatomically complex cases.

### 4.3 Risk Factors Affecting Seizure Control of SEEG‐3D RFTC in HH

We observed a significant correlation between SEEG‐3D RFTC outcomes in HH epilepsy and the degree of HH attachment disconnection and HH destruction, indicating that complete disconnection and ablation of the HH are essential for seizure control; even small amounts of residual tissue may lead to seizure recurrence, consistent with the findings of Kameyama et al. [15]. Furthermore, 80% of recurrences occurred within 6 months postoperatively, demonstrating that the therapeutic effect of SEEG‐3D RFTC is stable if destruction is complete.

HH‐associated epilepsy presents with completely different epileptogenic networks in GS and nGS [32]. The genesis of nGS symptoms may be secondary to networks outside the HH, whereby epileptiform discharges from HH cause GS and create abnormal connections with the hypothalamus. Consequently, new epileptogenic networks are independently formed, leading to nGS [33]. Therefore, the outcomes of GS and nGS were discussed separately in the present study.

This distinction is supported by both imaging and electrophysiological evidence. Studies using [18F]‐FDG PET and rs‐fMRI have shown that nGS patients often exhibit hypometabolism or altered connectivity in extra‐hypothalamic regions, including the cingulate cortex, anterior temporal lobe, and frontal operculum [34, 35]. SEEG recordings reveal that while GS typically originates focally within HH, nGS involves widespread or independent cortical onset, especially in medial frontal and temporal areas. Moreover, synchronous or sequential HH‐to‐cortex propagation further supports HH as a driver for secondary epileptogenesis [36]. These findings underscore that nGS is not merely an extension of GS but a distinct manifestation of HH‐related network evolution.

The percentage of HH ablation was the only important risk factor for GS. The laser ablation study by Boerwinkle et al. confirmed that, regardless of the HH size, favorable freedom from seizures was consistent if the laser ablation achieved complete destruction [37]. Our research achieved similar results by locally high‐density electrode arrangement and 3D RFTC.

On the contrary, the percentage of HH attachment ablation is more noteworthy for nGS. Although nGS often suggests a complex epileptic network, previous studies have confirmed that HH is an important node. Disconnecting the attachment still makes sense for the prognosis. Kameyama et al. suggested that disconnecting attachments could lead to favorable outcomes in patients with large HH [38].

Multiple studies have shown that LITT is quite efficient in treating HH, and our intention is not to replace the use of LITT with SEEG‐3D RFTC. However, compared with LITT, the implantation of SEEG electrodes and the use of 3D RFTC are more readily adaptable and cost‐efficient. Through this modified SEEG‐3D RFTC, we have also increased the ablation effect and improved the prognosis. We hope that SEEG‐3D RFTC will offer an alternate option to individuals in areas where LITT is not readily accessible, and this study can offer practical guidance for the application of SEEG‐3D RFTC.

## 5 Limitations

This study was a single‐center, retrospective analysis. No matched control group receiving alternative treatments (such as LITT or open surgery) was established, which limits the possibility of directly comparing the efficacy and safety among different surgical techniques. Therefore, future multicenter, prospective studies are needed to further validate the generalizability, long‐term effectiveness, and safety boundaries of this technique.

## 6 Conclusion

SEEG‐3D RFTC is an effective, safe, and stable minimally invasive treatment for HH‐related epilepsy and is especially suitable for promotion in areas where LITT is not available. Ablation of the HH and attachment site is essential for seizure control, and increasing the ablation ratio is the key to achieving seizure freedom. The concept of this study is also applicable to other minimally invasive procedures.

## Author Contributions

**Guoguang Zhao:** conceptualization and study design (lead). **Yongzhi Shan:** conceptualization, review, and editing (equal). **Penghu Wei:** conceptualization and review (equal). **Yang Dai:** writing – original draft (lead); formal analysis (lead); writing – review and editing (equal). **Yihe Wang:** writing – original draft (equal); writing – review and editing (equal). **Zesheng Li:** writing – original draft (equal); writing – review and editing (equal). **Josemir W. Sander:** review and editing. **Liankun Ren:** review and editing. **Xiaotong Fan:** review and data acquisition.

## Conflicts of Interest

The authors declare no conflicts of interest.

## Supporting information


**Table S1.** Seizure‐free survival after SEEG‐3D RFTC.
**Table S2.** GS‐free survival after SEEG‐3D RFTC.
**Table S3.** nGS‐free survival after SEEG‐3D RFTC.

## Data Availability

The data that support the findings of this study are available from the corresponding author upon reasonable request.
