# Supplementary material for: Optimized Stereo‐Electroencephalography‐Guided Three‐Dimensional Radiofrequency Thermocoagulation for Hypothalamic Hamartomas‐Related Epilepsy: A Single‐Center Experience in 69 Patients
Source: CNS Neurosci Ther. 2025 Jun 7;31(6):e70462. doi: 10.1111/cns.70462 (PMC12144596; doi:10.1111/cns.70462)
Supplement: Supplementary file 1 — Table S1. Seizure‐free survival after SEEG‐3D RFTC. Table S2. GS‐free survival after SEEG‐3D RFTC. Table S3. nGS‐free survival after SEEG‐3D RFTC. [file CNS-31-e70462-s001.docx]

**Supplementary Information**

**Optimized stereo-electroencephalography-guided three-dimensional radiofrequency thermocoagulation for** **hypothalamic hamartomas-related epilepsy: a single-center experience in 69 patients**

**Table S1. Seizure-free survival after SEEG-3D RFTC**

| Variables |  | Estimated seizure-free survival time (M) | 95% CI | Log-rank/Univariate Cox |
| --- | --- | --- | --- | --- |
| Sex | M | 53.856±5.707 | 42.670-65.042 | 0.421 |
|  | F | 65.280±7.020 | 51.521-79.039 |  |
|  |  |  |  |  |
| Age at surgery (years) | ≤7 | 52.139±8.039 | 36.383-67.895 | 0.765 |
|  | >7 | 57.818±6.695 | 44.695-70.941 |  |
|  |  |  |  |  |
| Age at seizure onset (years) | ≤2 | 55.899±6.145 | 43.855-67.944 | 0.443 |
|  | >2 | 64.407±6.707 | 51.261-77.553 |  |
|  |  |  |  |  |
| Duration of epilepsy (years) | ≤4.2 | 60.838±8.196 | 44.774-76.902 | 0.253 |
|  | >4.2 | 53.943±6.613 | 40.981-66.905 |  |
|  |  |  |  |  |
| HH classification | P | 51.867±10.230 | 31.816-71.917 | 0.665 |
|  | I | 47.000±6.496 | 34.267-59.733 |  |
|  | MU | 68.667±7.936 | 53.112-84.222 |  |
|  | MB | 53.565±7.836 | 38.207-68.923 |  |
|  |  |  |  |  |
| Precocious puberty | + | 55.429±10.604 | 34.645-76.213 | 0.543 |
|  | - | 55.921±5.103 | 49.920-69.923 |  |
|  |  |  |  |  |
| Maximum diameter | ≤15 mm | 65.500±5.386 | 54.944-76.056 | 0.371 |
|  | >15 mm | 54.333±6.990 | 40.632-68.034 |  |
|  |  |  |  |  |
| Previous surgery | + | 42.050±8.716 | 24.966-59.134 | 0.014 |
|  | - | 66.122±5.031 | 56.261-75.983 |  |
|  |  |  |  |  |
| Percentage of HH ablation |  |  |  | <0.001 |
|  |  |  |  |  |
| Percentage of HH attachment ablation |  |  |  | <0.001 |

SEEG-3D RFTC, stereo-electroencephalography-guided three-dimensional radiofrequency thermocoagulation; CI, confidence interval; M, male; F, female; P, parahypothalamic type; I, intrahypothalamic type; MB, mixed type with bilateral attachment; MU, mixed type with unilateral attachment; HH, hypothalamic hamartoma

**Table S2. GS-free survival after SEEG-3D RFTC**

| Variables |  | Estimated GS-free survival time (M) | 95% CI | Log-rank/Univariate Cox |
| --- | --- | --- | --- | --- |
| Sex | M | 64.234±5.338 | 53.771-74.697 | 0.862 |
|  | F | 69.909±6.825 | 56.531-83.287 |  |
|  |  |  |  |  |
| Age at surgery (years) | ≤7 | 54.662±8.281 | 38.432-70.893 | 0.260 |
|  | >7 | 73.321±5.410 | 62.718-83.925 |  |
|  |  |  |  |  |
| Age at GS onset (years) | ≤2 | 61.654±6.082 | 49.734-73.574 | 0.096 |
|  | >2 | 78.478±4.408 | 69.838-87.118 |  |
|  |  |  |  |  |
| Duration of GS (years) | ≤4 | 63.544±7.523 | 48.799-78.288 | 0.593 |
|  | >4 | 70.265±5.605 | 59.280-81.251 |  |
|  |  |  |  |  |
| HH classification | P | 70.679±8.941 | 53.153-88.204 | 0.725 |
|  | I | 44.857±7.246 | 30.656-59.058 |  |
|  | MU | 72.929±7.269 | 58.681-87.176 |  |
|  | MB | 64.635±7.305 | 50.317-78.953 |  |
|  |  |  |  |  |
| Precocious puberty | + | 61.286±10.022 | 41.643-80.928 | 0.289 |
|  | - | 69.572±4.697 | 60.365-78.779 |  |
|  |  |  |  |  |
| Maximum diameter | ≤15 mm | 73.203±4.981 | 63.439-82.966 | 0.079 |
|  | >15 mm | 56.435±7.434 | 41.865-71.005 |  |
|  |  |  |  |  |
| Previous surgery | + | 59.333±8.241 | 43.181-75.486 | 0.219 |
|  | - | 71.209±4.784 | 61.833-80.585 |  |
|  |  |  |  |  |
| Percentage of HH ablation |  |  |  | <0.001 |
|  |  |  |  |  |
| Percentage of HH attachment ablation |  |  |  | 0.001 |

SEEG-3D RFTC, stereo-electroencephalography-guided three-dimensional radiofrequency thermocoagulation; CI, confidence interval; M, male; F, female; P, parahypothalamic type; I, intrahypothalamic type; MB, mixed type with bilateral attachment; MU, mixed type with unilateral attachment; HH, hypothalamic hamartoma; GS, gelastic seizure

**Table S3. nGS-free survival after SEEG-3D RFTC**

| Variables |  | Estimated nGS-free survival time (M) | 95% CI | Log-rank/Univariate Cox |
| --- | --- | --- | --- | --- |
| Sex | M | 59.500±5.783 | 48.164-70.836 | 0.354 |
|  | F | 70.667±7.029 | 56.891-84.443 |  |
|  |  |  |  |  |
| Age at surgery (years) | ≤8 | 73.714±3.607 | 66.644-80.785 | 0.003 |
|  | >8 | 49.923±7.809 | 34.617-65.229 |  |
|  |  |  |  |  |
| Age at nGS onset (years) | ≤6 | 71.935±3.881 | 64.328-79.543 | 0.003 |
|  | >6 | 48.783±8.376 | 32.366-65.200 |  |
|  |  |  |  |  |
| Duration of nGS (years) | ≤2 | 72.030±4.936 | 62.355-81.705 | 0.049 |
|  | >2 | 50.000±8.069 | 34.184-65.816 |  |
|  |  |  |  |  |
| HH classification | P | 57.462±4.360 | 48.915-66.008 | 0.165 |
|  | I | 45.182±11.178 | 23.274-67.090 |  |
|  | MU | 71.846±7.917 | 56.328-87.364 |  |
|  | MB | 57.882±8.690 | 40.850-74.915 |  |
|  |  |  |  |  |
| Precocious puberty | + | 72.333±8.171 | 56.318-88.349 | 0.332 |
|  | - | 62.600±5.294 | 52.223-72.977 |  |
|  |  |  |  |  |
| Maximum diameter | ≤15 mm | 65.871±6.033 | 54.046-77.696 | 0.760 |
|  | >15 mm | 60.087±6.994 | 46.378-73.796 |  |
|  |  |  |  |  |
| Previous surgery | + | 42.125±9.728 | 23.058-61.192 | 0.002 |
|  | - | 73.526±4.368 | 64.965-82.088 |  |
|  |  |  |  |  |
| Percentage of HH ablation |  |  |  | 0.014 |
|  |  |  |  |  |
| Percentage of HH attachment ablation |  |  |  | <0.001 |

SEEG-3D RFTC, stereo-electroencephalography-guided three-dimensional radiofrequency thermocoagulation; CI, confidence interval; M, male; F, female; P, parahypothalamic type; I, intrahypothalamic type; MB, mixed type with bilateral attachment; MU, mixed type with unilateral attachment; HH, hypothalamic hamartoma; nGS, other types of seizures
